# Activation of the mechanosensitive ion channel MscL by mechanical stimulation of supported Droplet-Hydrogel bilayers

**DOI:** 10.1038/srep45180

**Published:** 2017-03-27

**Authors:** Kadla R. Rosholm, Matthew A. B. Baker, Pietro Ridone, Yoshitaka Nakayama, Paul R. Rohde, Luis G. Cuello, Lawrence K. Lee, Boris Martinac

**Affiliations:** 1 The Victor Chang Cardiac Research Institute, Lowy Packer Building, 405 Liverpool St, Darlinghurst, NSW 2010, Australia; 2 School of Medical Sciences, University of New South Wales, Kensington, NSW 2052, Australia; 3 Department of Cell Physiology and Molecular Biophysics, Center for Membrane Protein Research, Texas Tech University Health Sciences Center, Lubbock, TX 79430, USA; 4 St Vincent’s Clinical School, Darlinghurst, NSW 2010, Australia

## Abstract

The droplet on hydrogel bilayer (DHB) is a novel platform for investigating the function of ion channels. Advantages of this setup include tight control of all bilayer components, which is compelling for the investigation of mechanosensitive (MS) ion channels, since they are highly sensitive to their lipid environment. However, the activation of MS ion channels in planar supported lipid bilayers, such as the DHB, has not yet been established. Here we present the activation of the large conductance MS channel of *E. coli*, (MscL), in DHBs. By selectively stretching the droplet monolayer with nanolitre injections of buffer, we induced quantifiable DHB tension, which could be related to channel activity. The MscL activity response revealed that the droplet monolayer tension equilibrated over time, likely by insertion of lipid from solution. Our study thus establishes a method to controllably activate MS channels in DHBs and thereby advances studies of MS channels in this novel platform.

Droplet on Hydrogel Bilayers (DHBs) offer a new platform for studies of ion channels^1^. A key innovation in DHBs is that the lipid bilayer is oriented horizontally in the plane of the sample instead of vertically as in classical black lipid membranes^3^ and the recently developed droplet interface bilayers (DIBs)^1^. The horizontal orientation provides experimental versatility suitable for combining electrophysiology with microscopy techniques such as fluorescence resonance energy transfer or total internal reflection fluorescence microscopy that can be used for studies of ion channels^4^. In addition, DHBs allow the control of lipid composition in the bilayer, which is of particular importance for studies of mechanosensitive (MS) ion channels. Recent studies have presented the incorporation and mechanical activation of MS channels in the closely related DIBs^5^,^6^,^7^. However, the increased mechanical stability of planar supported bilayers, such as the DHB, makes them resistant to mechanical perturbation and thus the activation of MS channels in these systems has not been established. In this study we have employed the well-characterized EcMscL, the large conductance MS channel of *E. coli*, to establish for the first time the reconstitution and activation of a bacterial MS channel in DHBs.

During the last 25 years, it has been established that MscL and other prokaryotic MS channels are gated by the ‘force-from-lipids’ mechanism^8^,^9^, whereby channels are intrinsically mechanosensitive and thus able to directly detect and respond to membrane tension^10^. The crystal structure of MscL from *Mycobacterium tuberculosis*, which is homologous to EcMscL, revealed that the MscL channel is a homopentamer consisting of two transmembrane helices TM1 and TM2. The N- and C-terminal domains are located at the cytoplasmic side and a flexible loop is connecting the TM1 and TM2 helices at the periplasmic side of the bacterial cell membrane^11^,^12^. The gate of the MscL channel is formed by a ‘hydrophobic lock’, which consists of a cluster of hydrophobic residues in the channel pore. Introduction of polar or charged residues in this region makes the channel very sensitive to bilayer tension^13^,^14^. The closed to open transition of wild-type MscL is triggered by a membrane tension of ~9–10 mN/m^15^,^16^ and involves an iris-like expansion of the channel pentamer^17^,^18^,^19^. MscL is sensitive both to hydrophobic mismatch, meaning that thinner membranes drive expansion of the channel, and to asymmetry in the trans-bilayer tension profile induced by the insertion of conically shaped lipids such as lysophosphatidylcholine into one of the bilayer leaflets^17^,^20^.

To examine the mechanosensitivity of MscL in DHBs, we used in this study two gain-of-function (GOF) mutants of EcMscL, in which a Glycine residue in the hydrophobic gate of the protein was mutated to a glutamic Acid (MscL-G22E) or a serine (MscL-G22S), respectively (depicted in Fig. S3)^14^. We first optimized channel incorporation into the DHB using the spontaneously active MscL-G22E^21^. We then incorporated MscL-G22S, which is not spontaneously active but has a lower activation threshold than wild-type MscL^14^. This mutant was used to develop a method for controllably activating the channel by injecting buffer into the droplet, thereby stretching the inner DHB monolayer. Finally, we established a method for calculating the corresponding DHB tension and used MscL-G22S as a tension sensor to investigate how the DHB tension equilibrates over time upon injection of buffer. Our results comprise the first successful activation and characterization of MS channels in planar supported lipid bilayers. As this platform provides the capacity for parallel fluorescence and current measurements and the ability to control the lipid composition, this enables future investigations into the molecular mechanism of MS channels.

## Results

### Reconstitution of the spontaneously active MscL-G22E in DHBs

**Figure 1:**
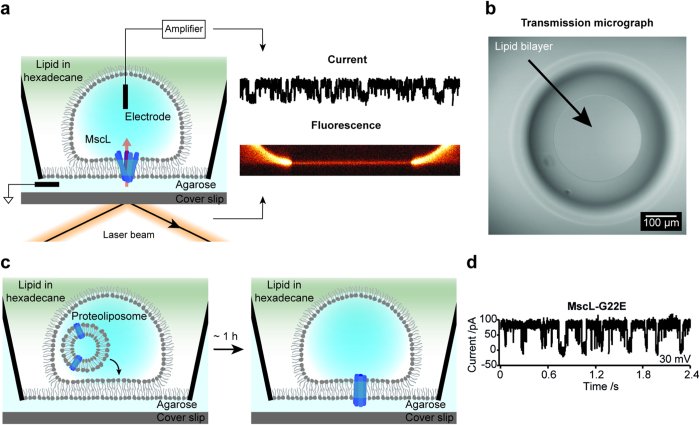
Successful reconstitution of MS ion channel MscL-G22E in DHBs. (**a**) Schematic showing the droplet hydrogel bilayer (DHB) (left). The DHB was formed in a well containing lipid in hexadecane (~10 g/L) between a lipid monolayer on an agarose-coated glass coverslip and a lipid monolayer surrounding an aqueous droplet. Incorporation of electrodes in the agarose and the droplet enabled measurements of currents from incorporated ion channels (right, top), while the horizontal orientation of the sample allowed imaging of the bilayer by fluorescence microscopy (right, bottom). (**b**) Top-view micrograph of the DHB captured by transmission microscopy. The inner white ring is the periphery of the bilayer and the larger blurred dark ring is the periphery of the droplet. The scale bar is 100 *μ*m. (**c**) Scheme depicting the reconstitution strategy. Fusogenic proteoliposomes containing the channel of interest were incorporated into the aqueous droplet (left). Within an incubation time of ~1 h the liposomes fused with the DHB thus incorporating the channel into the bilayer. (**d**) The single channel current recorded at +30 mV from MscL-G22E reconstituted into the DHB.

DHBs were formed as previously established^2^ between a planar lipid monolayer on a hydrophilic agarose surface and a lipid monolayer bordering an aqueous droplet (Fig. 1a) (see Methods). The bilayers were formed on a microscope coverslip allowing visualization of the bilayer by fluorescence light microscopy while the incorporation of electrodes in the agarose layer and the aqueous droplet, allowed measurements of ion channel currents (Fig. 1a, right). Bilayer integrity was confirmed by transmitted light microscopy (Fig. 1b) and by recording an increase in capacitance that is characteristic of bilayer formation (Supplementary Fig. S1 and Table S1). Bilayer fluidity was verified by Fluorescence Recovery After Photobleaching (FRAP) measurements (Supplementary Fig. S2 and Table S1).

As a model system we used DPhPC lipids (see structure in Supplementary Fig. S3a) as they have proven to reliably form very stable lipid bilayers^2^,^22^,^23^. To verify that we could successfully reconstitute MS ion channels into the DHBs, we first used a spontaneously active mutant of the channel, MscL-G22E^14^. The location of the mutation is depicted on the MscL WT crystal structure (PDB ID: 2OAR^11^) in Supplementary Fig. S3b. The protein was incorporated into small (~100 nm diameter) fusogenic proteoliposomes (see Methods) that subsequently were encapsulated in the aqueous droplet of the DHBs. Upon an incubation time of ~1 h the proteoliposomes fused with the bilayers facilitating MS channel reconstitution into the bilayers (Fig. 1c). Reconstitution of MscL-G22E resulted in spontaneous current traces representative of MscL gating (90 pA at +30 mV and 200 mM KCl)^21^, verifying that we could reconstitute MscL into DHBs (Fig. 1d).

### Activation of MscL-G22S in DHBs

**Figure 2:**
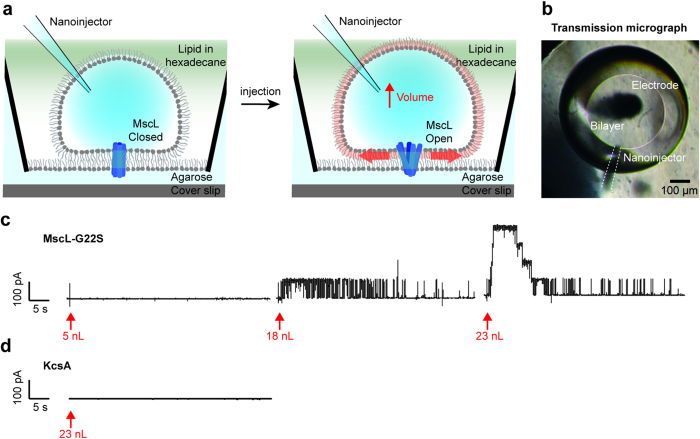
Activation of the MS ion channel MscL-G22S in DHBs. (**a**) Schematic showing the activation strategy. A nanoinjector equipped with a pulled glass pipette was inserted into the droplet (left). The injection of nanolitre volumes of buffer caused the droplet monolayer to stretch (right) thereby inducing an asymmetric tension in the DHB (red arrows), thus activating the channel. (**b**) Transmission micrograph of the bilayer viewed from the top. The inner white ring is the periphery of the bilayer and the larger black ring is the periphery of the droplet. An electrode (black) and nanoinjector (indicated by white dotted lines) were inserted into the droplet. The scale bar is 100 *μ*m. (**c**) Current traces, recorded at +30 mV in DHBs containing MscL-G22S, upon injection of 5 nL (left), 18 nL (middle) and 23 nL (right) experiment buffer. (**d**) Current traces recorded at +100 mV in DHBs containing resting or closed KcsA in a neutral pH buffer (pH = 7.4), upon the injection of 23 nL experiment buffer.

Next, we developed a method for MS channel activation in DHBs. Since MscL is activated by asymmetric stretching and bending of the lipid bilayer^17^,^24^, we sought to activate the channel by selectively stretching the droplet leaflet of the DHB by increasing the droplet volume. This was achieved by injecting nanolitre volumes of buffer into the droplet using a nanoinjector equipped with a pulled glass pipette tip (Fig. 2a). We reconstituted the MscL mutant, MscL-G22S, which is ~30% easier to open than wild-type MscL (see Supplementary Fig. S3c)^14^. A transmission micrograph of the droplet viewed from the top, with both an electrode (black) and a nanoinjector (white dotted lines) inserted, is depicted in Fig. 2b. As hypothesized, above a certain threshold the addition of buffer into the droplet induced tension in the DHB, which was sufficient to activate MscL-G22S (Fig. 2c, middle), resulting in current traces representative of MscL gating (90 pA at +30 mV and 200 mM KCl)^21^. Subsequently, increasing the injection volume increased the number of active channels (Fig. 2c).

To verify that the activation response was selective for MS channels we used the well-characterized proton-gated ion channel, KcsA^25^,^26^. The channel was spontaneously active in droplets containing acidic buffer (pH = 4) resulting in current recordings of multiple channels opening and closing (Supplementary Fig. S4a) with conductance levels representative of KcsA gating (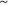
~20 pA at +100 mV and 300 mM KCl)^27^. Applying stepwise 20 mV increments of voltage (from −100 mV to 100 mV) allowed us to generate a current vs. voltage (IV) plot that was consistent with equivalent plots of KcsA determined from patch clamp experiments^27^ (Supplementary Fig. S4b and c). Unlike MscL, however, injecting volumes of buffer into droplets containing either resting or closed (pH = 7.4) or active (pH = 4) KcsA, did not influence KcsA activity (Fig. 2d and Supplementary Fig. S5, respectively).

### Quantification of DHB tension

**Figure 3:**
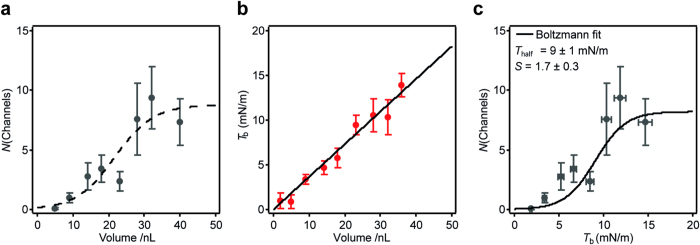
Quantification of MscL-G22S sensitivity to bilayer tension in DHBs. (**a**) The maximum number of activated channels observed right after the injection, *N*(Channels), as a function of the volume of injected buffer. Each data point is the average and s.e.m. of at least 4 droplets (see Supplementary Table S2). (**b**) The quantified DHB tension, *T*_b_, as a function of the volume of injected buffer, revealing a linear dependency within the range of volumes investigated. Each data point is the average and s.e.m. of at least 3 droplets. (**c**) The number of active channels as a function of quantified DHB tension. The fit of a Boltzmann function (black solid line) allowed us to quantify the tension at which half of the channels are open, *T*_half_ = 9 ± 1 mN/m, and the slope, *S* = 1.7 ± 0.3. Each data point is the average and s.e.m. of at least 4 droplets (see Supplementary Table S2).

To calculate the DHB tension, *T*_b_ (mN/m), induced in the bilayers upon buffer injection, we combined activation data from many droplets (see Supplementary Table S2). This allowed us to plot the number of activated MscL-G22S channels as a function of injected volume (Fig. 3a). The increase in droplet monolayer tension upon extension or compression can be calculated as Δ*T*_m_ = *K*_*A*_ · (Δ*A/A*)^5^, where *K*_*A*_ = 120 mN/m is the area modulus of elasticity of a DPhPC monolayer^28^, *A* is the droplet surface area before injection, and Δ*A* is the increase in droplet surface area upon injection. The increase in bilayer tension is given as *T*_b_ = Δ*T*_m_ · cos(*θ*), where *θ* is the angle between the droplet and agarose monolayer at the point of contact between the two monolayers (see Supplementary Fig. S6a). From fluorescence micrographs of the DHBs we recorded *θ* = 19 ± 4° for ~100 nL equilibrated droplets (Supplementary Fig. S6a), which was within the range (5°–50°) previously quantified for droplet-droplet bilayers^5^. Since cos(19°) = 0.99 ≈ 1, we estimated the basal bilayer tension in our system *T*_b_ ≈ Δ*T*_m_. We quantified *A* and Δ*A* from transmission micrographs of the droplet and bilayer captured before and after injection, respectively (Supplementary Fig. S6b). Plotting *T*_b_ versus injected volume revealed a linear dependency within the range of volumes investigated (Fig. 3b) allowing us to plot the number of active channels versus DHB tension (Fig. 3c). We fit a Boltzmann distribution function to the data to quantify the tension at which half of the channels are open, *T*_half_ = 9 ± 1 mN/m, and the slope, *S* = 1.7 ± 0.3 m/mN.

For comparison we also quantified the tension sensitivity of MscL-G22S in DPhPC bilayers using patch fluorometry as previously described^29^,^30^. MscL-G22S was incorporated into DPhPC liposomes labeled with 0.1% fluorescent lipid (rhodamine-PE), which were subsequently used in the patch clamp experiments. This allowed us to visualize DPhPC liposome patches in the patch pipette using confocal fluorescence microscopy (Supplementary Fig. S8a), while simultaneously applying stepwise increases in bilayer pressure and recording the resulting ion channel currents (Supplementary Fig. S8b). Following a previously established analysis method^29^ and using Laplace’s equation, we quantified bilayer tension from the patch curvature (Supplementary Fig. S8a) allowing us to plot the open probability, *P*_o_, of MscL-G22S as a function of bilayer tension (Supplementary Fig. S8c). Boltzmann fits to the experimental data yielded the tension *T*_half_ = 11.1 ± 0.5 mN/m, and the slope, *S* = 1.4 ± 0.1 m/mN (Supplementary Fig. S8c). Both parameters were in good agreement (within 20%) of the tension and slope quantified in DHBs.

### Characterization of the DHB tension equilibration process

**Figure 4:**
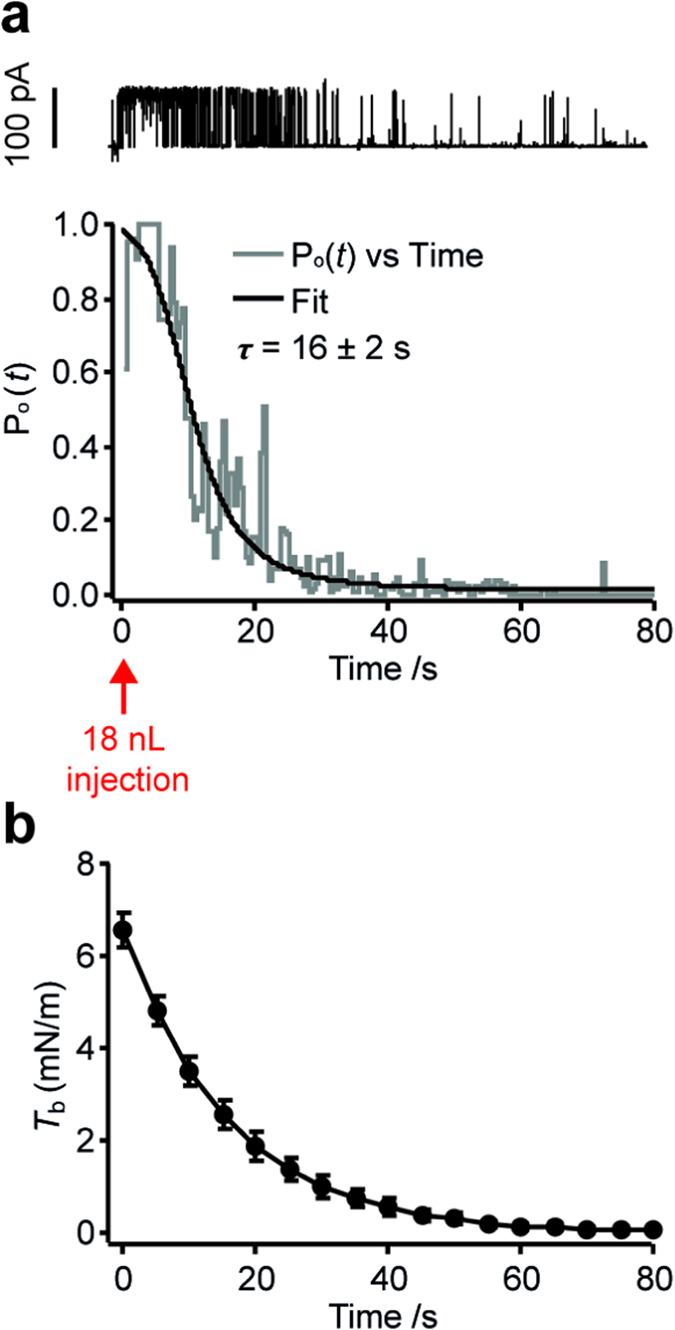
Time-dependent tension equilibration in the DHB. (**a**) Top: Current trace of channel activity, recorded at +30 mV in DHBs containing MscL-G22S, upon the injection of 18 nL experiment buffer into the droplet. Bottom: The corresponding plot of channel open probability, *P*_o_(*t*), as a function of time taken as the average between three individual experiments (see Supplementary Fig. S9). Assuming an exponential tension decay, we can fit a model to the data (solid black line) and extract the bilayer tension decay rate, *τ* = 16 ± 2 s. (**b**) The DHB tension, quantified in (**a**), as a function of time upon injection of 18 nL buffer. The error bars are propagated from the s.e.m. on the initial tension quantified in Fig. 3 (6.6 ± 0.4 mN/m) and the s.d. on the extracted decay rate.

From the traces of current in Fig. 2c it is evident that the injection-induced DHB tension decreased over time, most likely due to the stressed lipid monolayer equilibrating with free lipid in hexadecane (~10 g/L) through processes such as the Marangoni effect^31^. To address how the bilayer tension propagates over time we used MscL-G22S as a tension sensor. We quantified the open probability as a function of time, *P*_o_(*t*), for single MscL-G22S channels after injection of 18 nL experiment buffer (Supplementary Fig. S9 and Fig. 4a). The adsorption of free lipid into the droplet monolayer can be seen as a two-state process (the lipid being free in solution or inserted in the monolayer). Thus we assume an exponential decay of DHB tension vs. time, which concurs with previous studies of bilayer tension relaxation in excised patches^32^. By substituting an exponential decay for the bilayer tension in the Boltzmann equation we can then describe the channel open probability vs. time as follows:


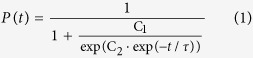


where *τ* is the exponential time constant of the bilayer tension decay and C_1_ and C_2_ are constants, C_1_ = exp(*T*_half_/*S*) and C_2_ = exp(*T*_0_/*S*), where *T*_half_ and *S* is the Boltzmann fitting parameters quantified for MscL-G22S above and *T*_0_ is the bilayer tension at the time *t* = 0. Physically, the constants C_1_ and C_2_ represent the free energy difference ΔG in units of *k*_B_*T* between the closed and open state of an MS channel^33^ and are characteristic of the MS channel reconstituted into the DHB. Fitting Eq. (1) to the data (Fig. 4a, black line) allowed us to extract the bilayer tension decay time constant, *τ* = 16 ± 2 s and plot the exponential DHB tension propagation over time (Fig. 4b). Note that the equilibration is about ten times slower in the DHB than quantified in excised patches^32^ and DIBs^34^. This difference can be rationalized by a number of differences between the assays, including the membrane geometry, the membrane area (~100,000 *μ*m^2^ and ~6 *μ*m^2^ for DHBs (Supplementary Table S1) and excised patches^35^, respectively) and the equilibration process (lipid insertion in the DHB; monolayer sliding in excised patches and DIBs).

### DHBs as a model system for studying mechanosensation

**Figure 5:**
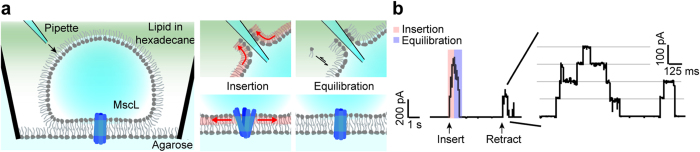
Activation of MscL-G22S by mechanical stimulation of “touch-sensitive” droplets. (**a**) Scheme of the experimental apparatus (left). Inserting a pulled glass pipette into the droplet causes the lipid monolayer to creep up the hydrophilic glass surface thereby stretching the monolayer and opening the channel (middle). The tension is rapidly equilibrated by insertion of lipid from the hexadecane solution (right). (**b**) Current traces recorded at +30 mV in DHBs containing MscL-G22S. The mechanical stimulus caused a burst of channel activity, followed by rapid channel closing as the membrane tension equilibrated again.

The fact that the DHBs equilibrate the tension over time makes them a convenient model system for studying mechanosensation in whole organisms, where multiple mechanisms are in place to relax the membrane upon a mechanical perturbation^32^,^36^. To mimic such a situation in the DHB system, we reconstituted MscL-G22S as described above and generated a controlled, reproducible mechanical stimulus, by piercing the droplet monolayer with a glass pipette (Fig. 5a), while recording the activation response of the ion channel. Figure 5b shows current responses of the channel as the pipette is repeatedly inserted and subsequently removed from the droplet monolayer. The measurements revealed a “touch-sensitivity” of the droplets resulting in a burst of channel activity upon each mechanical stimulus (Fig. 5b, red shading) followed by equilibration (Fig. 5b, blue shading).

## Discussion

Reconstitution of ion channels in artificial bilayers, including planar bilayers and liposomes, has for many years served as the pertinent reductionist approach in studies of ion channels in cell membranes. These systems enable not only the study of channel function in isolation from other cell membrane components, but also the investigation of the effects of various lipids on this type of membrane protein. The latter is in particular important for studies of the MS class of ion channels given that the function of many MS channels is intimately linked to their lipid environment. Several examples that immediately come to mind include MS channels of bacteria and archaea, such as MscL, MscS, MscMJ and MscCG^10^,^15^,^29^,^30^,^37^ and of mammalian cells, including Piezo1, TREK-1 and TRAAK^38^,^39^,^40^,^41^.

The recently developed DHBs^1^,^2^,^22^,^42^ offer several advantages for investigating MS channels as compared to traditional bilayer methods, such as the patch clamp setup. These benefits include easier and faster sample preparation, the option to perform multiple experiments in parallel, and the possibility to form lipid bilayers of asymmetric lipid composition that mimic the asymmetry of biological membranes. However, even if recent studies have presented the incorporation of MS channels in the closely related DIBs^5^,^6^,^43^, to date the incorporation and activation of MS channels in DHBs have not been established.

In this study we report the successful reconstitution and activation of the MS channel MscL in DHBs. As the prototypical MS channel, MscL is well suited to test new bilayer techniques for their feasibility to study MS channels. We used two MscL GOF mutants, MscL-G22E and MscL-G22S, and incorporated them in stable and long-lasting DHBs made from DPhPC lipids. The MscL-G22E mutant was spontaneously active in DHBs, which served as a proof-of-principle for the successful reconstitution of an MS channel into DHBs. Similar to Leptihn *et al*.^44^ we introduced the proteoliposomes during DHB formation, however the time-scale of channel incorporation (~1 h) was slower than reported in this study (immediate protein insertion). We believe this is because we introduce proteoliposomes in the aqueous solution of the droplet rather than from the agarose side. In this case channel insertion is limited by the liposome diffusing through the droplet volume and thus the channels are most likely incorporated by vesicle fusion with the bilayer *after* DHB formation.

Next we developed a method for activating MS channels in DHBs, by systematically generating asymmetric bilayer tension. For these experiments we used the MscL-G22S GOF mutant, which is not spontaneously active like the G22E GOF mutant but has a threshold of tension activation that is twice as low as the wild-type MscL^14^, (in azolectin lipid ∼6 mN/m and ∼12 mN/m^16^, respectively). We selectively stretched the droplet monolayer by injecting increasing volumes of buffer into the droplet, thus creating step-wise increments in asymmetric bilayer tension, which activated increasing numbers of reconstituted channels (Fig. 2). This is to our knowledge the first demonstration of controllable activation of an MS channel in a supported planar lipid bilayer. As a control we used the same protocol and applied it to reconstituted KcsA ion channels^25^, whose activity, unlike that of MscL-G22S, did not change upon injection of buffer (Supplementary Fig. S5).

Adapting a previously established method^5^ we were able to quantify the tension sensitivity of MscL-G22S in DPhPC DHBs (*T*_half_ = 9 ± 1 mN/m and *S* = 1.7 ± 0.3 m/mN). The results were in good agreement (within 20%) with the sensitivity quantified using a previously published method (see Supplementary Fig. S8) thus confirming our tension estimation. Interestingly, the channel required a higher tension to gate in DPhPC bilayers (9–11 mN/m) as compared to azolectin bilayers (~6 mN/m), likely due to the increased rigidity of DPhPC bilayers (*K*_*A*_ = ~120 mN/m)^28^ as compared to azolectin bilayers (*K*_*A*_ = ~45.5 mN/m)^29^.

A notable property of the DHBs is their ability to equilibrate the induced tension over time (see Fig. 4). We suggest that the main contribution to this equilibration is lipid adsorption from the highly concentrated (~10 g/L) solution of lipid in hexadecane, which has been observed in similar setups to happen within minutes^45^. This property makes the droplets interesting for investigating how MS channels adapt to relaxation/remodeling processes in the membrane upon mechanical stimuli^32^,^36^. As an example, we utilized the fact that the droplets were “touch sensitive” to mimic mechanosensation by poking the droplet with a nanopipette (Fig. 5) thus generating a transient stimulus of membrane tension. We envision that the systematic study of an MS channel response as a function of pipette position or poking speed could provide significant insights into the nature of bilayer tension propagation and channel adaptation in mechanosensation.

In conclusion, the successful demonstration of MscL mechanosensitivity in DHBs represents a significant contribution to the study of mechanosensory transduction and MS ion channels. Our study establishes a unique platform, which allows the complete control of each component of the system, the lipid composition, the mechanical stimuli involved, and the specific MS channels under study and is thus well suited to test specific pharmacological agents modifying the channel activity. On a broader scale, it also allows the visualization of fluorescently labeled MS proteins when any of these variables are changed, thus making it for the first time possible to investigate the structure-activity relationship of MS channels at the single-molecule level. We envisage that this can be used in the future for characterization of any MS channel including e.g. Piezo ion channels, whose malfunction in recent years has been shown to underlie a number of mechanopathologies^46^,^47^,^48^.

## Methods

### Materials

#### Chemicals

Chloroform (Cat. No. 650498), hexadecane (Cat. No. H6703), agarose (Cat. No. A9414), KCl (Cat. No. P5405), MgCl_2_ (Cat. No. M8266) and Azolectin (Cat. No. P5638) were purchased from Sigma-Aldrich^®^. 4-(2-hydroxyethyl)-1-piperazineethanesulfonic acid (HEPES, Cat. No. A1069) was purchased from PanReac AppliChem. n-Dodecyl-β-D-Maltopyranoside (DDM, Cat. No. D310) was purchased from Anatrace. 1,2-dioleoyl-*sn*-glycero-3-phosphoethanolamine-N-(lissamine rhodamine B sulfonyl) (ammonium salt) (rhodamine-PE, Cat. No. 810150) and 1,2-diphytanoyl-*sn*-glycero-3-phosphocholine (DPhPC, Cat. No 850356) were purchased from Avanti^®^ Polar lipids. Sodium Hypochlorite (4% Bleach, True Blue Chemicals, Caringbah, Australia), Triton X-100 (Anatrace, Ohio).

#### Materials

Silver wire (100 *μ*m diameter, Cat. No. 348783 and 1.5 mm diameter, Cat. No. 348759) was purchased from Sigma-Aldrich^®^. Microscope coverslips (borosilicate glass, 24 mm × 40 mm, thickness no. 1 (0.13–0.17 mm, Cat. No. CS2440100) were purchased from Menzel-Gläser. Extrusion filters (PC Membrane, 0.1 *μ*m, Cat. No. 610005) were purchased from Avanti^®^ Polar lipids. Bio-Beads SM-2 (Cat. No. 152–8920) were purchased from Bio-Rad. The droplet-hydrogel bilayer device and the droplet incubation chamber were custom-made from PMMA substrate at Oxford University as described by Leptihn *et al*.^2^.

### Mutagenesis

MscL-G22S expression construct was made from a wild-type MscL 3.1 construct using a *QuikChange* (Agilent Technologies, Santa Clara, CA, USA) thermal polymerase type site directed mutagenesis strategy.

### Protein expression and purification

#### MscL-G22E

MscL-G22E was prepared using cell-free expression as previously described^21^.

#### MscL-G22S

MscL-G22S was expressed in BL-21 (DE3) (Novagen) *E. coli*, grown at 37 °C to OD_600_ 0.8 and induced with 1 mM IPTG for 3 h. A retrieved cell pellet was then suspended in PBS with ~0.02 mg/mL DNase (Sigma DN25) and 0.02% PMSF (Amresco M145) and broken with a TS5/48/AE/6 A cell disrupter (Constant Systems) at 31,000 psi at 4 °C. Cell debris was removed by centrifugation (12,000 × g 15 min 4 °C) and then membranes were pelleted for 45000 RPM in a Type 45 Ti rotor (Beckman) for 3 h at 4 °C. Membrane pellets were solubilized in PBS with 8 mM DDM overnight at 4 °C. The solubilisation was clarified with a 12000 × g 20 min 4 °C centrifugation, and then bound to cobalt sepharose (Talon^®^, 635502, Clontech) followed by washes with PBS supplement with 15 mM Imidazole (Sigma, 56750) and then eluted with 500 mM imidazole PBS. The concentration of imidazole was decreased by using a 100 kDa Amicon-15 centrifugal filter unit (Merck Millipore) with DDM PBS. Protein concentration was estimated by polyacrylamide electrophoresis with SimplyBlue™ (LC6065, Thermo Fisher) staining.

#### KcsA

KcsA was expressed in freshly made C41 DE3 competent cells (Lucigen Madison, WI) in LB media Luria-Bertani (LB) broth medium, supplemented with 0.5% glycerol (as a chemical chaperone) 0.2% glucose, 0.4 mg/ml ampicillin, 0.1 mM (IPTG) and 10 mM BaCl_2_ at 30 °C for 20 h, and purified as recently described^49^. In brief, KcsA was extracted with the 1.5% Triton (Anatrace, Ohio) 50 mM Tris-Cl + 1 M KCl (Buffer A) and protease inhibitors for 1 h at room temperature. The solubilized material was spun down at 100,000 g and KcsA was purified by metal-chelated chromatography and gel filtration.

### Droplet-Hydrogel Bilayer (DHB) formation

The Droplet-Hydrogel bilayers were prepared as described by Leptihn *et al*.^2^. In brief, a solution of DPhPC in hexadecane was prepared by transferring 190 *μ*L of DPhPC in chloroform (50 mg/mL) to a glass vial. The solvent was removed by rapid swirling under nitrogen followed by at least 30 min in a vacuum desiccator to form a solvent free lipid film. 1 mL of hexadecane was added to the lipid film. Vortexing was followed by 10 min sonication to produce a solution of DPhPC in hexadecane (9.5 mg/mL).

#### Device preparation

The DHB device (PMMA) contains 16 1-mm-diameter wells, each designed to contain a DHB, and on the underside a microfluidic channel surrounding each well. The device was prepared by spin-coating (Laurell Technologies Corporation^®^) 140 *μ*L of agarose solution onto a plasma-cleaned (Harrick Plasma, PDC-32G) glass coverslip (0.75% wt/vol, 90 °C, 4000 rpm, 30 s) producing a thin layer (<300 nm) of agarose on the surface^2^. The device was positioned on top of the agarose-coated coverslip and sealed by addition of agarose through the microfluidic channel (3.25% wt/vol in experiment buffer).

#### Bilayer formation

A droplet incubation chamber was filled with lipid in hexadecane (9.5 mg/mL, 200 *μ*L) and four ~200 nL droplets (experiment buffer) were pipetted into each groove in the chamber. Each well on the device was also filled with lipid in hexadecane (9.5 mg/mL, 60 *μ*L). The device and the droplets were incubated for 25 min, allowing monolayer formation, before the droplets were transferred to each of the 16 wells, spontaneously forming bilayers upon touch down on the surface (after ~30 min incubation).

### Protein reconstitution in DHBs

#### Proteoliposome formation

A lipid film of azolectin was prepared in a glass vial by dissolving 2 mg azolectin in chloroform followed by evaporation of the organic solvent under nitrogen flow. The lipid film was rehydrated by adding 1 mL surfactant-containing buffer (200 mM KCl, 5 mM HEPES, 1 mM DDM, pH 7.4), followed by vortexing and 15 min sonication. The lipid sample was then extruded through a filter (100 nm pore size), using a lipid vesicle extruder from AVESTIN, followed by addition of protein (KcsA, MscL-G22E or MscL-G22S) in a 1:250 protein:lipid ratio. After 1 h incubation at 4 °C on a rotary table, biobeads (~10 mg) were added to remove surfactant. To ensure complete surfactant removal and proteoliposome formation the biobeads were exchanged four times over 24 h (after incubation periods of 2 h, 2 h, 15 h and 2 h).

#### Protein reconstitution in DHBs

The proteoliposome solution was diluted 100× in the experiment buffer and used for droplet formation. Protein activity was observed ~1 h after bilayer formation.

### Imaging of DHBs

Transmission images of the bilayers were recorded on an inverted light microscope (Nikon Diaphot) using a CFI LWD objective (20×; NA 0.4; Nikon). Images were collected through the eyepiece using a digital camera (Canon Powershot S3 IS) with a microscope eyepiece adaptor. Fluorescence images of DHBs, labeled by incorporating a fluorescently labeled lipid, rhodamine-PE (0.1%), were recorded on an inverted confocal microscope (Zeiss LSM 700) using a low magnification (20×; NA 0.4; Carl Zeiss) or high magnification (63×; NA 1.15; Carl Zeiss) objective.

#### Fluorescence Recovery After Photobleaching (FRAP)

We utilized FRAP experiments to ensure that lipids were freely diffusing in the DHB. We bleached a circular area (*R* ≈ 13 *μ*m) of the fluorescently labeled DHB and recorded the fluorescence recovery as a function of time.

### Electrophysiological recordings in DHBs

Electrodes (Ag/AgCl) were prepared as previously described^2^ using silver wires with diameters of 1.5 mm and 100 *μ*m for the ground electrode and the droplet electrode, respectively, which were immersed in sodium hypochlorite (4%) overnight to form a AgCl coating. The ground electrode was inserted into the agarose reservoir (3.25% wt/vol). The droplet electrode was coated in agarose (3.25% wt/vol in experiment buffer) and inserted into the droplet upon bilayer formation using a micromanipulator. Both electrodes were connected to a Bilayer Clamp Amplifier (BC-535, Warner Instruments). The current was filtered at 1 kHz and acquired at 5 kHz with a Digidata 1440 A interface using pCLAMP 10 acquisition software (Molecular Devices, Sunnyvale, CA) and analyzed. Current traces for figures have subsequently been smoothed using a box algorithm (10 points).

### Protein activation in DHBs

**MscL-G22E** was spontaneously active in the DHBs. **KcsA** was activated by diluting the proteoliposome sample in a low pH buffer (300 mM KCl, 5 mM HEPES, pH = 4.0) before monolayer incubation. **MscL-G22S** was activated by injecting nL volumes (5, 9, 18, 23, 32 or 36 nL) of experiment buffer (200 mM KCl, 5 mM HEPES, pH = 7.4) into the droplet (23 nL/s) using a nanoinjector fitted with a glass micropipette (Nanoliter 2000, World Precision Instruments). At large injections (>30 nL) the DHB area decreased as a function of droplet volume (see Fig. S7), suggesting that the induced DHB tension does not equilibrate all the way back to its initial level upon injection. Consequently, we used only one injection per droplet.

### Patch Fluorometry

#### Proteoliposome formation

A lipid film of DPhPC:rhodamine-PE (99.9:0.1 wt:wt) was prepared in a glass vial by dissolving the lipids in chloroform (2 mg total mass) followed by evaporation of the organic solvent under nitrogen flow. The lipid film was rehydrated in 200 *μ*L surfactant-containing buffer (B1) (0.5 mM KCl, 5 mM HEPES, 1 mM DDM, pH 7.4), followed by vortexing and 15 min sonication. MscL-G22S was added at a protein:lipid ratio of 1:100. The mixture was transferred to a 10 mL falcon tube and B1 buffer was added to a total volume of 3 mL. After 1 h incubation at RT on a rotary table, biobeads (~10 mg) were added to remove surfactant. To ensure complete surfactant removal the biobeads were exchanged four times over 24 h (after incubation periods of 2 h, 2 h, 15 h and 2 h). The resulting sample was spun down in an ultracentrifuge (Beckman Optima LE-80K, USA) (30 min, 40000 RPM). After removing the buffer the lipid pellet was rehydrated in 60 *μ*L D/R buffer (300 mM KCl, 5 mM HEPES, pH 7.4), spotted onto a clean microscope glass slide (20 *μ*L per spot) and dehydrated in a vacuum desiccator for at least 6 h. Each proteoliposome spot was rehydrated in 60 *μ*L D/R buffer for at least 3 h before the patch clamp experiment.

#### Patch clamping

The patch clamp experiment was carried out and analyzed using standard procedures as previously described^30^,^50^ in patch buffer (200 mM KCl, 40 mM MgCl_2_, 5 mM HEPES, pH = 7.4).

#### Fluorescence imaging

Inside-out excised DPhPC patches, containing MscL-G22S and fluorescently labeled with rhodamine-PE, were imaged using a Zeiss LSM 700 confocal microscope using a long working distance water immersion objective (63×; NA 1.15; Carl Zeiss). A 555 nm laser line was used to excite the rhodamine-PE and the emission was detected using a long-pass 560 nm filter. To visualize the liposome patch at the confocal microscope, the pipette tip was bent with a microforge (Narishige; MF-900) to make it parallel with the bottom of the patch chamber.

## Additional Information

**How to cite this article:** Rosholm, K. R. *et al*. Activation of the mechanosensitive ion channel MscL by mechanical stimulation of supported Droplet-Hydrogel bilayers. *Sci. Rep.* **7**, 45180; doi: 10.1038/srep45180 (2017).

**Publisher's note:** Springer Nature remains neutral with regard to jurisdictional claims in published maps and institutional affiliations.

## Supplementary Material

Supplementary Information
