# Supplementary material for: Activation of the mechanosensitive ion channel MscL by mechanical stimulation of supported Droplet-Hydrogel bilayers
Source: Sci Rep. 2017 Mar 27;7:45180. doi: 10.1038/srep45180 (PMC5366917; doi:10.1038/srep45180)
Supplement: Supplementary Information [file srep45180-s1.pdf]

Supplementary Information

Activation of the mechanosensitive ion channel MscL by mechanical stimulation of supported Droplet-Hydrogel bilayers

Kadla R. Rosholm[1], Matt Baker[2], Pietro Ridone[1], Yoshitaka Nakayama[1], Paul R. Rohde[1], Luis G. Cuello[4], Lawrence Lee[1,2] and Boris Martinac[1,3,*]

[1] The Victor Chang Cardiac Research Institute, Lowy Packer Building, 405 Liverpool St, Darlinghurst, NSW 2010, Australia, [2], School of Medical Sciences, University of New South Wales, Kensington, NSW 2052, Australia, [3]St Vincent's Clinical School, Darlinghurst, NSW 2010, Australia, [4]Department of Cell Physiology and Molecular Biophysics, Center for Membrane Protein Research, Texas Tech University Health Sciences Center, Lubbock, TX 79430

* Correspondence to b.martinac@victorchang.edu.au

Supplementary text

Characterization of DHBs. DHBs prepared from 100% DPhPC lipids or DOPC:DOPG (PC:PG) lipids at molar ratio 90:10 were characterized by quantifying 1) the average bilayer area, *A*, 2) the specific capacitance, *C*, of the bilayer, and 3) the diffusion coefficient, *D*, of an incorporated fluorescently labeled lipid (Supplementary Table S1).

A unilamellar lipid bilayer separating two conducting liquids functions as a capacitor. Consequently, applying a triangular voltage across the membrane results in a characteristic square wave capacitive current, *I*(t).

$$I(\mathrm{t}) = C\frac{dV(\mathrm{t})}{d\mathrm{t}}$$

Since bilayer capacitance is proportional to bilayer area, *A*, bilayer formation can be followed by recording the increase in *I*(t) over time (Supplementary Fig. S1). Upon bilayer formation we can measure the area *A* by imaging the bilayer by transmission microscopy (Fig. 1b) and quantify the specific capacitance, *C*, of the bilayer as follows:

$$C = \frac{\varepsilon\varepsilon_0 A}{d}$$

where ε and ε_0 are the dielectric coefficients of lipid and vacuum, respectively and *d* is the thickness of the hydrophobic bilayer core. The quantified capacitance of the two bilayer compositions was identical, ~0.6 μF/cm^2 (Supplementary Table S1), and within the range (0.3 – 0.8 μF/cm^2) of previously published values quantified in similar systems[1, 2].

Finally, we quantified lipid diffusion by Fluorescence Recovery After Photobleaching (FRAP) experiments (Supplementary Fig. S2). We labeled the droplet monolayer by incorporating 0.1 mole% DOPE-Rhodamine into the droplet monolayer. By bleaching a defined area of the bilayer (grey box in Supplementary Fig. S2a) and quantifying the fluorescence recovery as lipids diffuse back into that area (Supplementary Fig. S2b) by normalizing the recovery curve as previously established[3] and fitting a previously published model[4] we extracted the time

constant of diffusion, τ_D, and calculated the diffusion coefficient, D, as follows:

$$D = \frac{R^2}{4\tau_D}$$

where R is the radius of the bleached area. The quantified diffusion (Supplementary Table S1) was twice as fast in the PC:PG bilayer than in the DPhPC bilayer (~8 $\mu m^2/s$ and ~4 $\mu m^2/s$, respectively). This observation is in general agreement with the carbon chains of DPhPC lipids being more sterically hindered, due to the methyl groups (Supplementary Fig. S3a), as compared to the carbon chains of DOPC and DOPG. Interestingly, comparing the diffusion coefficient obtained in the PC:PG bilayer to previously published diffusion coefficients published for DOPC[5] indicates that the mobility in agarose-supported bilayers is similar to that of free-standing bilayers ($D = 7.8 \pm 0.8$ $\mu m^2/s$) rather than solid-supported bilayers ($D = 3.1 \pm 0.3$ $\mu m^2/s$).

MscL mutants. We used in this study two previously characterized MscL GOF mutants, MscL-G22E and MscL-G22S. In both mutants a Glycine residue in the pore of the protein was mutated to either a Glutamic Acid (MscL-G22E) or a Serine (MscL-G22S) (see Supplementary Fig. S3b). Introducing a hydrophilic amino acid in this position was previously shown to disrupt the 'hydrophobic lock' in the channel thereby decreasing its tension sensitivity in the case of MscL-G22S or rendering it spontaneously active in the case of MscL-G22E[6] (see Supplementary Fig. S3c).

Reconstitution and activation of KcsA. As a negative control we used the well-characterized proton-gated ion channel, KcsA[7-9]. The channel was reconstituted as established for MscL in droplets containing acidic buffer (pH = 4), resulting in current recordings of multiple channels opening and closing (Supplementary Fig. S4a). Histograms of the current amplitudes are depicted in Supplementary Fig. S4b. Applying stepwise 20 mV increments of voltage (from -100 mV to 100 mV) allowed us to generate a current vs. voltage (IV) plot that was consistent

with equivalent plots of KcsA gating measured in patch clamp experiments[10] (Supplementary Fig. S4c). We tested whether buffer injection into the droplet could influence KcsA activity by inserting a nanoinjector equipped with a pulled glass pipette tip into a droplet containing active KcsA at pH = 4 (Fig. 2a) and injecting 23 nL of a matching buffer (Supplementary Fig. S5). Unlike MscL stretching the droplet monolayer influenced neither the amount of active KcsA channels nor the frequency of channel openings. At pH = 7.4 the channel was in the closed state both with and without injections (Fig. 2d).

Quantification of the angle between the two monolayers. The angle, θ, at the point of contact between the two monolayers was quantified by incorporating 0.1 mole% fluorescently labeled lipid, rhodamine-PE, into the droplet monolayer and capture fluorescent images in the z-direction. Since the agarose monolayer is parallel with the surface of the sample, the angle was extrapolated by measuring the angle between the droplet monolayer and the surface at the point of monolayer separation (Supplementary Fig. S5a).

Quantification of MscL-G22S tension sensitivity in DPhPC using patch fluorometry. MscL-G22S was incorporated into DPhPC liposomes labeled with 0.1% rhodamine-PE. This allowed us to visualize the DPhPC patch using confocal fluorescence microscopy (Supplementary Fig. S7a), while simultaneously applying stepwise increases in bilayer pressure and recording the resulting ion channel currents (Supplementary Fig. S7b). As previously established[11] we quantified the radius of membrane curvature, r, from the fluorescence images and calculated membrane tension, T, using Laplace's law; $T = rP/2$, where P is the pressure applied to the patch pipette causing the membrane patch to form a hemisphere (Supplementary Fig. S7a). This allowed us to plot the open probability, P_o, of MscL-G22S as a function of bilayer tension (Supplementary Fig. S7c).

Supplementary Figures

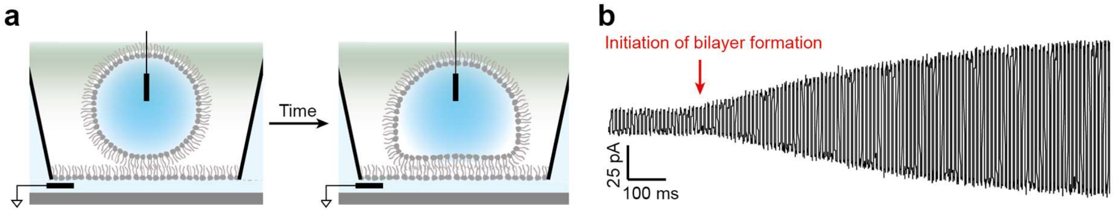

Figure S1. Recorded increase in capacitance characteristic of bilayer formation. (a) Scheme of bilayer formation. An aqueous droplet surrounded by a lipid monolayer touches down onto a planar lipid monolayer formed on an agarose surface (left). After incubation (~25 min) a bilayer forms between the droplet and the agarose monolayers (right). (b) By applying a triangular voltage across the membrane we recorded the characteristic square wave capacitive current, which increases as the bilayer is forming. From the bilayer area, *A*, we quantified the specific capacitance, *C*, of the bilayer (see Table S1).

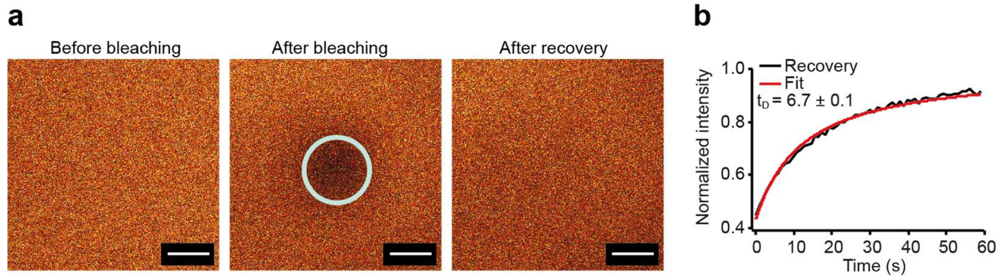

Figure S2. Verification of bilayer fluidity by Fluorescence Recovery After Photobleaching (FRAP) experiments. (a) Fluorescence micrographs of a DPhPC DHB, labeled with rhodamine-PE (see Methods), before bleaching (left), after bleaching (middle) and after fluorescence recovery (right). The scale bar is 20 μm. (b) The normalized intensity within the bleached area (grey ROI in (a)) as a function of time (black). By fitting an exponential model[4] we could extract the characteristic lipid diffusion time, τ_D, (see Table S1).

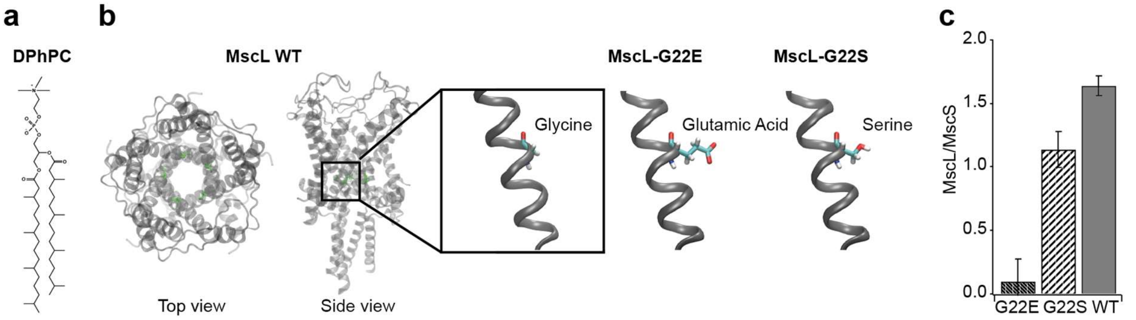

Figure S3. Structure of DPhPC lipid and MscL mutants. (a) Chemical structure of DPhPC. (b) Top and side view of the crystal structure of WT MscL (PDB ID: 2OAR[12]) with a Glycine residue highlighted in green, which was mutated in the two GOF mutants to either a Glutamic Acid (MscL-G22E) or a Serine (MscL-G22S). (c) The previously published tension sensitivity, given as the ratio between the patch clamp pressures necessary for gating MscL and MscS, plotted for MscL-G22E, -G22S and WT[6].

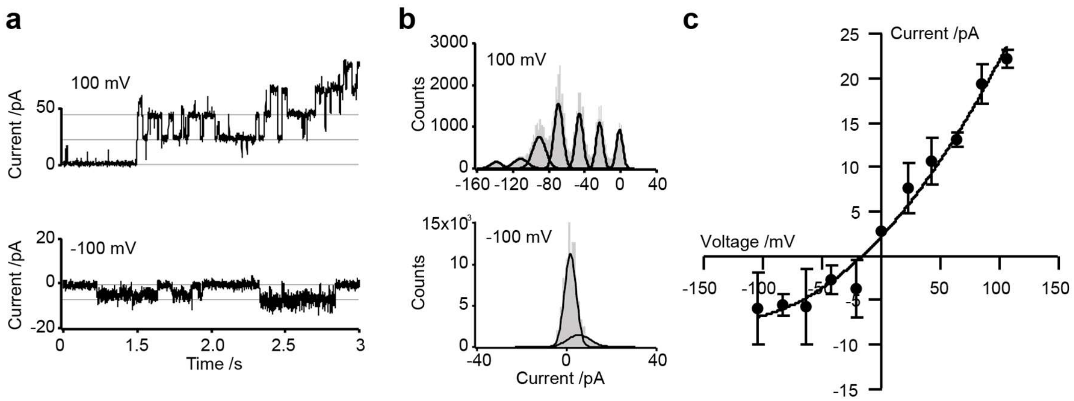

Figure S4. Activation of KcsA in DHBs. (a) Current traces recorded at +100 mV (top) and -100 mV (bottom) in DHBs containing KcsA in a low pH buffer (300 mM KCl, pH = 4). (b) Amplitude histograms of KcsA currents recorded at +100 mV (top) and -100 mV (bottom). (c) Current versus voltage plot for KcsA recorded in DHBs at pH = 4.

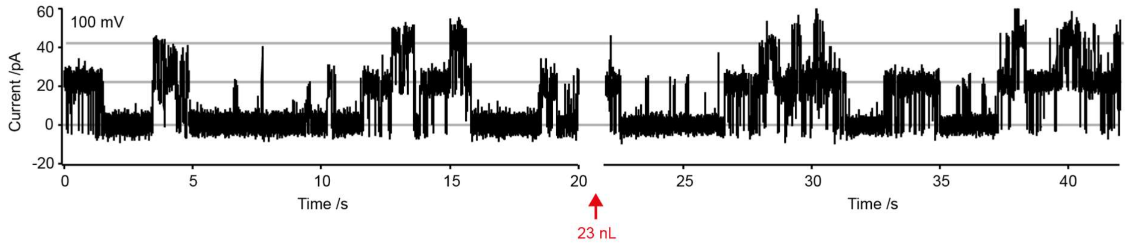

Figure S5. Buffer injection does not influence KcsA activity. Current traces recorded at +100 mV in DHBs containing active KcsA in a low pH buffer (300 mM KCl, pH = 4) before (left) and after (right) injection of 23 nL experiment buffer.

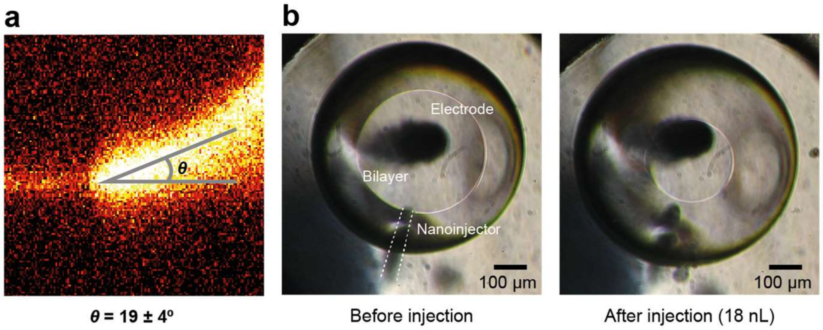

Figure S6. Estimation of DHB tension. (a) The contact angle, θ, between the droplet and agarose monolayer was quantified from fluorescence micrographs, captured along the z-axis. The average and s.e.m. of four DHBs yielded an angle of 19 ± 4°. (b) Transmission micrographs of the bilayer (white inner rim) and droplet (black outer rim) before (left) and after (right) injection of 18 nL experiment buffer. The bilayer area decreases due to the increased tension in the droplet monolayer, which has a vector component perpendicular to the bilayer.

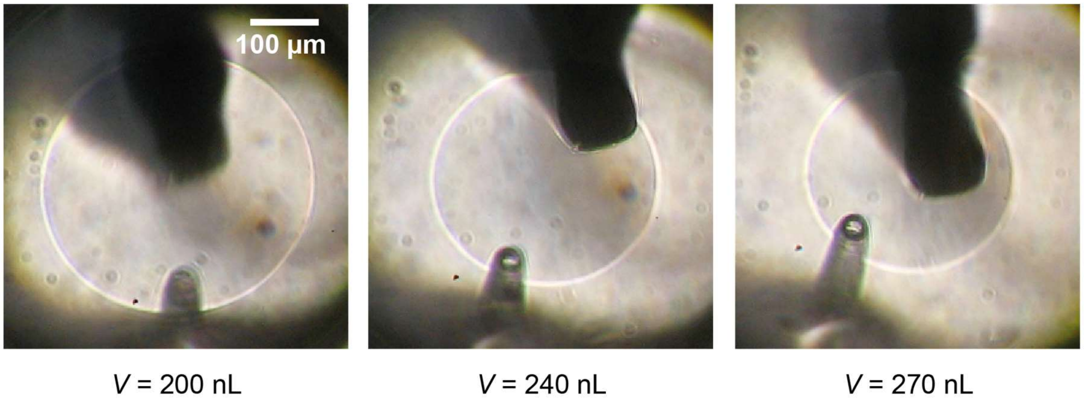

Figure S7. The equilibrated DHB area decreases as a function of droplet volume. At large injections (> 30 nL) the equilibrated bilayer area decreases as a function of droplet volume (V). Micrographs of the same DHB before injection (V = 200 nL) (left), after injection of 40 nL (V = 240 nL) (middle) and after injection of additional 30 nL (V = 270 nL) (right). The micrographs are captured 10 min after injection ensuring that the area was completely equilibrated.

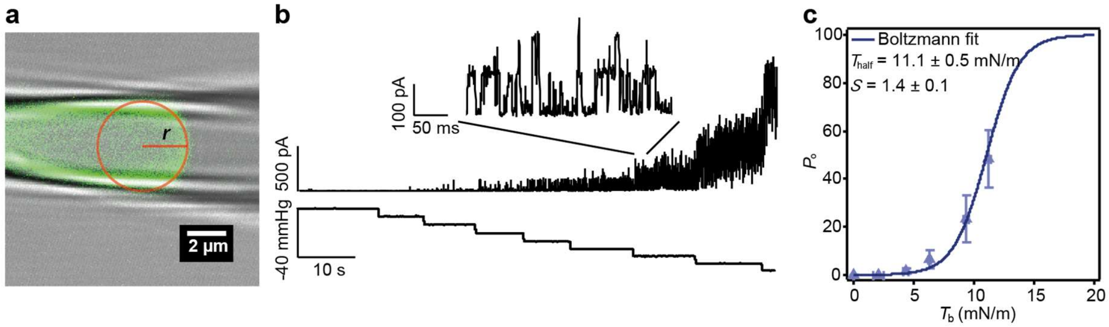

Figure S8. Validation of the DHB tension estimation by patch fluorometry. (a) Overlay of the transmission and fluorescence micrograph of a DPhPC membrane patch labeled with rhodamine-PE (green) in a pulled glass pipette. The application of negative pressure to the patch pipette caused the membrane to form a hemisphere. Following a previously established analysis method[11] we quantified the radius of membrane curvature, *r*, of the membrane patch (indicated by the red circle), which was subsequently used to quantify the membrane tension using Laplace's law. (b) Current (top) and pressure (bottom) traces recorded at +30 mV in DPhPC patches containing MscL-G22S. (c) Plot of the MscL-G22S open probability, P_o, versus bilayer tension, T_b. Each data point is the average and s.e.m. of three different experiments. Fitting a Boltzmann function (blue solid line) allowed us to extract the $T_{half} = 11.1 \pm 0.5$ mN/m, and the slope, $S = 1.4 \pm 0.1$ m/mN.

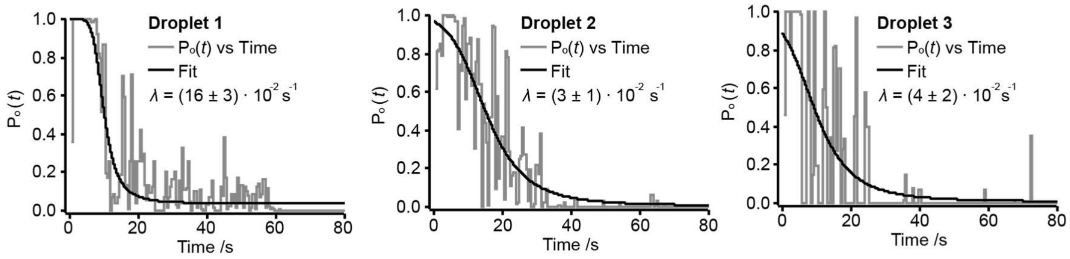

Figure S9. Time-dependent tension equilibration in the DHB of three individual droplets. Channel open probability, $P_o(t)$, as a function of time upon the injection of 18 nL experiment buffer in three different droplets. Assuming an exponential tension decay, we can fit a model to the data (solid black line) and extract the bilayer tension decay rate, $\lambda = (16 \pm 3) \cdot 10^{-2}$ s^{-1}, $\lambda = (3 \pm 1) \cdot 10^{-2}$ s^{-1} and $\lambda = (4 \pm 2) \cdot 10^{-2}$ s^{-1}, for droplet 1, 2 and 3, respectively. The difference between the quantified rates are likely due to small variations in droplet volume, and consequently an average of the three traces was used to estimate the tension equilibration in DHBs.

Lipid composition	*A* /μm²	*N*	*C* /μF cm⁻²	*N*	*D* /μm² s⁻¹	*N*
PC:PG 90:10	115000 ± 6000	11	0.64 ± 0.08	8	7.8 ± 0.9	5
DPhPC	96000 ± 2000	17	0.64 ± 0.05	11	4 ± 1	5

Table S1. Characterization of DHBs prepared from PC:PG or DPhPC lipids. From left to right is listed the average bilayer area, *A*, bilayer capacitance, *C*, and lipid diffusion coefficient, *D*, for PC:PG DHBs (top row) and DPhPC DHBs (bottom row). *N* is the number of experiments and the error is the s.e.m..

Injected volume /nL	$N_{Droplets}$	$N_{Channels}$ (min)	$N_{Channels}$ (max)	$N_{Channels}$ (ave +/- SEM)
5	16	0	1	0.06 ± 0.06
9	6	0	3	1.0 ± 0.5
14	4	0	5	2.7 ± 1.1
18	7	1	10	3.4 ± 1.2
23	8	0	7	2.4 ± 0.8
28	10	1	29	7.5 ± 3.0
32	8	2	22	9.4 ± 2.6
40	16	1	28	7.3 ± 1.9

Table S2. Number of droplets and activated MscL-G22S channels at increasing volumes of injected buffer. The columns contain from the left: the injected volume, the number of droplets ($N_{Droplets}$), and the minimum ($N_{Channels}$(min)), maximum ($N_{Channels}$(max)) and average ($N_{Channels}$) number of MscL-G22S channels observed. The error is the s.e.m. between the individual droplets.

References

1. Schuster, B., Pum, D., Braha, O., Bayley, H. & Sleytr, U.B. Self-assembled alpha-hemolysin pores in an S-layer-supported lipid bilayer. *Biochim. Biophys. Acta* **1370**, 280-288 (1998).
2. Florin, E.L. & Gaub, H.E. Painted supported lipid membranes. *Biophys. J.* **64**, 375-383 (1993).
3. Phair, R.D. *et al.* Global nature of dynamic protein-chromatin interactions in vivo: three-dimensional genome scanning and dynamic interaction networks of chromatin proteins. *Mol. Cell. Biol.* **24**, 6393-6402 (2004).
4. Soumpasis, D.M. Theoretical analysis of fluorescence photobleaching recovery experiments. *Biophys. J.* **41**, 95-97 (1983).
5. Przybylo, M. *et al.* Lipid diffusion in giant unilamellar vesicles is more than 2 times faster than in supported phospholipid bilayers under identical conditions. *Langmuir* **22**, 9096-9099 (2006).
6. Yoshimura, K., Batiza, A., Schroeder, M., Blount, P. & Kung, C. Hydrophilicity of a single residue within MscL correlates with increased channel mechanosensitivity. *Biophys. J.* **77**, 1960-1972 (1999).
7. Cuello, L.G., Cortes, D.M., Jogini, V., Sompornpisut, A. & Perozo, E. A molecular mechanism for proton-dependent gating in KcsA. *FEBS Lett.* **584**, 1126-1132 (2010).
8. Cortes, D.M. & Perozo, E. Structural dynamics of the Streptomyces lividans K+ channel (SKC1): oligomeric stoichiometry and stability. *Biochemistry* **36**, 10343-10352 (1997).
9. Tilegenova, C., Vemulapally, S., Cortes, D.M. & Cuello, L.G. An improved method for the cost-effective expression and purification of large quantities of KcsA. *Protein Expr. Purif.* **127**, 53-60 (2016).

10. Meuser, D., Splitt, H., Wagner, R. & Schrempf, H. Exploring the open pore of the potassium channel from Streptomyces lividans. *FEBS Lett.* **462**, 447-452 (1999).

11. Nomura, T. *et al.* Differential effects of lipids and lyso-lipids on the mechanosensitivity of the mechanosensitive channels MscL and MscS. *Proc. Natl. Acad. Sci. USA* **109**, 8770-8775 (2012).

12. Chang, G. Structure of the MscL Homolog from Mycobacterium tuberculosis: A Gated Mechanosensitive Ion Channel. *Science* **282**, 2220-2226 (1998).
